# Process evaluation of a person-centred outcome measures-based quality improvement program in a hospital-based palliative care in mainland China

**DOI:** 10.1007/s11136-025-03997-w

**Published:** 2025-05-27

**Authors:** Yunyun Dai, Jinfeng Ding, Yongyi Chen, David Holloway, Junchen Guo, Yu Cheng, Claire E Johnson

**Affiliations:** 1 Faculty of Science, Medicine and Health, University of Wollongong, 239 Squires Way, Wollongong, NSW 2522 Australia; 2 School of Nursing, Guilin Medical University, Guilin, Guangxi China; 3 Xiangya School of Nursing, Central South University, Changsha, Hunan China; 4 The Affiliated Cancer Hospital of Xiangya School of Medicine, Hunan Cancer Hospital, Central South University, Changsha, Hunan China; 5 Electronic Persistent Pain Outcomes Collaboration (ePPOC), Faculty of Science, Medicine and Health, University of Wollongong, Sydney, NSW Australia; 6 School of Nursing, Faculty of Science, Medicine and Health, University of Wollongong, Wollongong, NSW Australia; 7 Jinxin Geriatric Hospital, Chengdu, Sichuan China; 8 Palliative Aged Care Outcomes Program, Faculty of Science, Medicine and Health, University of Wollongong, Wollongong, NSW Australia

**Keywords:** Person-centred outcome measures, Quality improvement program, Hospital-based palliative care, Process evaluation, Normalization process theory

## Abstract

**Purpose:**

This study aimed to understand Chinese palliative care clinicians’ experience in integrating the Palliative Care Outcome Collaboration (PCOC) model into their clinical practice and to learn lessons for sustainability.

**Methods:**

An explanatory mixed-methods study was conducted. Combing semi-structured in-depth interviews with palliative care clinicians guided by Normalization Process Theory (NPT) with analysis of clinical documents to examine implementation outcomes. Qualitative data were analysed using a combination of inductive and deductive content analysis, quantitative data were presented using frequencies and percentages.

**Results:**

Twelve out of 16 clinicians participated in this study. Six months post-implementation, over half reported successful integration of the model into their unit. Implementation outcomes indicated strong clinician adherence, with all inpatients assessed and 75% of clinicians participating. Eleven sub-themes were identified within the NPT constructs, explaining the mechanisms contributing to its success and failure. These included clinicians’ perception of the model’s value and effectiveness (Coherence), accurate assessment and effective use of results and quality reports (Collective Action), and a supportive network that fully incorporated clinicians’ views (Cognitive Participation), and aligning the PCOC model with existing workflows, reducing redundant tools, and streamlining documentation (Reflexive Monitoring).

**Conclusions:**

To effectively implement a PCOMs-based quality improvement program into routine clinical practice, a “top-down” followed by a “bottom-up” implementation approach is recommended. Effectively utilizing the program to achieve its value and fit into existing workflows without adding unnecessary workload could ensure its sustainability. Furthermore, in countries and regions where palliative care is emerging, priorities should focus on enhancing clinicians’ knowledge, self-efficacy, and supporting multidisciplinary collaboration.

**Supplementary Information:**

The online version contains supplementary material available at 10.1007/s11136-025-03997-w.

## Abstract (plain language summary)

Palliative care in Mainland China is still in its infancy, and the absence of quality improvement programs within this field highlights a significant gap in care. Person-centred Outcome Measures (PCOMs)-based quality improvement programs have been widely used to manage quality in healthcare systems. The Australian Palliative Care Outcome Collaboration (PCOC) model presents an exemplar framework that has shown substantial improvements in the quality of palliative care. To address the gap in the field of palliative care in Mainland China, we planned to adapt and integrate the PCOC model at a Chinese cancer hospital-based palliative care unit. After six months of implementation, we evaluated how the PCOC model was being used, what worked, how it worked and why it worked in this specific context. Therefore, this study aimed to understand the experience of Chinese palliative care clinicians in integrating the PCOC model and to learn lessons for sustainability and future scale-up. Our findings offer valuable insights into the factors contributing to the success or the challenges of integrating a complex intervention into routine clinical practice. We believe these insights could help bridge the gap between research and practical application, particularly in less developed palliative care countries and regions where quality improvement is urgently needed.

**Supplementary Information:**

The online version contains supplementary material available at 10.1007/s11136-025-03997-w.

## Introduction

Person-centred outcomes measures (PCOMs) are standardised and validated tools that include both patient-reported and proxy-reported outcome measures [1]. These tools have emerged as an essential part of person-centred care, enabling healthcare providers to tailor interventions to individual needs and improve the quality of care delivery [2]. In palliative care, where understanding and addressing the holistic needs of patients is paramount, PCOMs play a critical role in facilitating, guiding decision-making and monitoring outcomes that matter most to patients and their families/carers [3, 4].

Globally, PCOMs have been widely adopted in palliative care practice, particularly in high-income countries. For instance, the Australian Palliative Outcome Collaboration (PCOC) model presents an exemplar framework that has demonstrated statistically and clinically significant improvements in patient outcomes [5]. Similarly, in the United Kingdom and the United States, PCOMs have been integrated into healthcare systems to support value-based care models [6, 7]. Despite the success, the implementation of PCOMs in low- and middle-income countries and regions, including Mainland China, remains limited. This gap is particularly pronounced in palliative care, where infrastructure, workforce training and public awareness are still developing [8].

One of the most well-established frameworks for integrating PCOMs into palliative care is the PCOC model, a national initiative developed in Australia [9]. Funded by the Australian Government Department of Health and Aged Care, PCOC is designed to evaluate and support continuous improvement of palliative care outcomes based on ongoing point-of-care assessment of individual patients’ and carers’ palliative care needs. PCOC supports continuous improvement through a quality feedback loop which includes routine assessment of patients’ and carers’ needs using standardized tools (Supplementary Fig. 1) [9]. Additionally, PCOC provides bi-annual quality reports to participating services, allowing them to compare clinical outcomes with national, consensus-based benchmarks and to identify areas for improvement. These efforts are supported by improvement facilitators across the country and benchmarking workshops (Supplementary Fig. 2).

In Mainland China, palliative care is in its early stage of development [10], with a lack of standardized framework for quality evaluation and improvement [11]. The integration of evidence-based models, such as the PCOC, offers a promising avenue for addressing these challenges. Compared with other PCOMs program, such as the Palliative care Outcome Scale (POS) and the Integrated Palliative care Outcomes Scale (IPOS) [12], PCOC demonstrates how assessments can be translated into an actionable care plan, quality reporting, feedback loops and continuous improvement [9]. Due to its success, the PCOC model is being adopted in many other countries and regions around the world, including Ireland, Singapore and Taiwan [9, 13]. Therefore, we adapted and integrated the PCOC model at a cancer hospital-based palliative care unit in Mainland China.

To scale-up the PCOC model in China, it is essential to move beyond merely focusing on “an effectiveness evaluation”. We need to understand what works, how it works and why it works in this specific context by conducting a thorough process evaluation. Process evaluation is an integral component in designing and testing of complex interventions as it helps identify the key elements and the potential mechanisms that contribute to its success or failure [14–16]. This understanding is essential for replicating interventions in different settings, and bridging the gap between efficacy and practical implementation, ensuring the interventions are both effective, sustainable and scalable in diverse settings [14].

In this study, we conducted a process evaluation to understand the dynamics of integrating the PCOC model into routine clinical practice in a cancer hospital-based palliative care unit. The aims were to: (i) understand how the PCOC model was implemented, (ii) assess the outcomes of its implementation, (iii) explore the experience of palliative care clinicians in integrating the PCOC model, and (iv) learn lessons for sustainability and future scale-up of PCOC model in China.

## Methods

### Implementation context and process

The PCOC model was formally implemented in a 22-bed palliative care unit at a cancer hospital in Mainland China from 1st June 2023. This unit was staffed by 12 nurses and 4 doctors. Before formal implementation, all clinicians attended a 45-minute on-site PCOC fundamentals education session, and a thorough assessment of the contextual factors was conducted (reported in Palliative & Supportive Care), which informed modifications to the PCOC model and the development of tailored implementation strategies. Two modifications were made: the assessment frequency was adjusted to daily, instead of the original PCOC recommendation of “at least once a day” based on changing in plan of care; and the quality report schedule was changed from biannual to quarterly. The five PCOC tools were integrated into the hospital’s Electronic Medical Records system with the support of the IT department.

A dedicated implementation team, including the unit clinical director, the head nurse and the director of the nursing department, oversaw the process. An external facilitator was on-site during the first week of implementation to address any immediate questions about the PCOC model. Subsequently, monthly meetings were held between clinicians and the external facilitators to discuss challenges and monitor adherence. Every three months, a quality report was produced, accompanied by a PCOC advanced education session delivered by the external facilitator. A quality improvement plan was developed collaboratively by the nurse manager and the external facilitator based on the reports.

A detailed description of the full range of implementation strategies is presented in Supplementary Table 1, using the terminology of the Expect Recommendations for Implementation Change (ERIC) framework [17, 18] and Proctor’s guidelines for reporting the implementation strategies [19].

### Study design

This study employed an explanatory mixed methods design to conduct a process evaluation, integrating quantitative outcome evaluation with qualitative process evaluation to provide a comprehensive understanding of PCOC implementation. Quantitative data were used to assess the implementation outcomes of the PCOC model, while the qualitative component, guided by Normalization Process Theory (NPT) [20], examined how the PCOC model was implemented and the experiences of palliative care clinicians in integrating the PCOC model. Normalization Process Theory was chosen due to its use of four interconnecting constructs (coherence, cognitive participation, collective action, and reflexive monitoring) that help explain how complex interventions become routine practice and is widely used to close the gap between research and practical application [21]. These quantitative data were later interpreted alongside qualitative interview findings to provide a more comprehensive understanding of how and why the PCOC model was integrated into practice.

### Participants

This process evaluation was conducted in December 2023, six months after its implementation. All 16 clinicians from the palliative care unit were invited to participate in in-depth, semi-structured individual interviews.

### Data collection

#### Interviews

The interview guide, developed based on the NPT and finalized by the research team (Supplementary Table 2), was pilot tested by authors X1 and X2, both skilled in semi-structured in-depth interviews, with two nurses from another department. Interviews were conducted face-to-face in the palliative care unit’s meeting room, with one conducted online. To ensure a quiet and uninterrupted environment, only the interviewer and interviewee were present. All interviews were audio or video recorded.

### Clinical document records

Quantitative data were collected through a systematic review of clinical documents to evaluate the PCOC implementation in terms of “Reach”, “Adoption” and “Fidelity” [22, 23] (Table 1). Daily PCOC assessment records, along with the information of clinicians conducting the rating, were extracted from the Electronic Medical Records for the period of June to November 2023. These data were retrieved in December 2023. All data were de-identified and replaced with a study ID number before being transferred to the research team for analysis.

**Table 1:** The index for the PCOC model implementation outcomes evaluation

Implementation outcomes	Description	Evaluation index
Reach (Individual level)	The absolute number, proportion, and representativeness of individuals who are willing to participate in a given initiative, intervention, or program[23].	• The percentage of palliative care patients assessed by the PCOC tools
Adoption (Multiple setting and staff levels)	The absolute number, proportion, and representativeness of settings and intervention agents (people who deliver the program) who are willing to initiate a program[23].	• The percentage of the palliative care clinicians in the palliative care unit who actively participated in the PCOC model (By checking the PCOC assessment records and medical records every three months)
Fidelity (Individual and setting levels)	Refers to the extent to which a policy or intervention is delivered as intended by its developers and in line with the programme model[22].	• Tools assessment o The assessment frequency using the five PCOC tools o The rate of data item completion
		• Quality report and improvement plan o The number of quality feedback report o The number of improvement plan

### Data management and analysis

Audio and video recordings were transcribed in Chinese, checked for accuracy and anonymized before being imported into NVivo version 12 for data analysis [24]. A hybrid inductive-deductive analysis was employed to explore clinicians experience with PCOC implementation, the inductive approach allowed themes to emerge directly from the data, while the deductive approach ensured alignment with NPT.

Initially, two authors (X1, a PhD candidate in palliative care with five years’ experience in qualitative study; and X2, a PhD candidate in palliative care with three years’ experience in qualitative study, both proficient in Chinese and English) thoroughly familiarized themselves with the records and transcripts. Together, they identified sub-themes from one transcript to ensure consistency, then independently coded the remaining transcripts. Codes with similar meanings were grouped into sub-themes, reflecting key emerging patterns. These sub-themes were iteratively refined into overarching themes that represented key aspects of PCOC implementation, similar sub-themes were categorized into broader themes. Several meetings were held to discuss and compare the themes between the two coders until a consensus was reached.

After the initial inductive coding, the finalized themes were then mapped to the four constructs of the NPT using a deductive approach. The results were tabulated into the NPT coding manual, developed by May et al., for qualitative research [25]. The transcripts were in Chinese, only the sub-themes and themes were translated into English (by X1). A senior researcher proficient in both Chinese and English (X3, PhD in palliative care), with extensive experience in qualitative research reviewed the translations for accuracy to ensure the quality of data analysis and reduce researcher bias.

All steps of the data analysis were clearly documented for transparency and the results were confirmed with the research team. The combination of inductive and deductive approaches allowed us to remain open to new insights emerging from the data while also situating our findings within the established theoretical framework of the NPT, thereby enhancing the depth and rigor of our analysis [26]. Quantitative data was presented using frequencies and percentages.

### Ethical approval and consent to participate

This study was performed in line with the principles of the Declaration of Helsinki. It was led by the University of Wollongong, with data collection taking place in the palliative care unit at the Hunan Cancer Hospital. As such, ethical approval was obtained from the University of Wollongong (2022/160) and the Hunan Cancer Hospital (KY2022217).

Prior to participation, all clinicians were provided with a Participate Information Sheet outlining the study’s purpose, methods, involved activities, data management procedures, and potential risks and benefits. Participants were informed that participation was voluntary and they could withdraw from the study at any time until their data was de-identified and included in the research database. After that point, data removal was longer possible due to the removal of identifying information. Informed, written consent was obtained from all study participants before the commencement of the study.”

## Results

### Characteristics of participants

Twelve out of sixteen clinicians from the palliative care unit participated in the study. Four who did not participate were on leave or declined. Most participants were female (*n* = 10, 83.3%), with an average age of 36.1 years (ranging from 27 to 45 years) and 1–10 years of experience in palliative care. Twelve individual semi-structured interviews were conducted and averaged 39.5 min.

### Implementation outcomes

We evaluated the implementation outcomes of the PCOC model using three primary indices: reach, adaptation, and fidelity.

*Reach* Throughout the six months of PCOC implementation, all palliative care inpatients (*n* = 355, 100%) were assessed using the PCOC tools.

*Adoption* Initially, all clinicians used the PCOC model, but participation dropped to 75% (12 out of 16 clinicians) due to staff leave or working part time.

*Fidelity* Clinicians demonstrated strong adherence to the Chinese PCOC protocol, conducting daily assessments. Two quality feedback reports and two improvement plans were produced as proposed. Reports indicated that only 2.2% of PCOC daily assessment data were missing (71/3205 daily assessments).

### Implementation mechanisms

Eleven sub-themes within the NPT framework were identified from the interview data to explain the mechanisms contributing to the successes and challenges of integrating the PCOC model (see Supplementary Table 3).

### Coherence (Understanding and making sense of PCOC Implementation)

Coherence involves the sense-making work that palliative care clinicians engage in, both individually and collectively, when integrating the PCOC model into routine clinical practice [20]. Two themes were identified within this construct.

### Sub-theme 1 distinctiveness of of PCOC compared to other quality initiatives

After six months of the PCOC implementation, senior clinicians identified significant differences between the PCOC model and traditional methods such as satisfaction surveys and standard hospital policies for quality improvement, and they recognized the relative advantage of the PCOC model, particularly in its emphasis on person-centred holistic care. A doctor noted, “*We used to rely on satisfaction surveys to assess care quality. Now*,* with PCOC*,* we can identify what we are doing well*,* and which areas need improvement and further enhancement. Additionally*,* it clearly determines patients’ palliative care phase*,* and also quantifying the care quality.*”

### Sub-theme 2 perceived value of the PCOC model in patient care

The PCOC model’s value was widely recognized among clinicians, including less experienced staff. They reported it could assist them in providing timely and personalized care, making medical decisions, developing care plans, and facilitating communication. A nurse expressed, “*The PCOC assessment helps identify patients’ symptoms*,* allowing me to relieve their discomforts timely.*”

### Cognitive participation (Engagement and commitment to Implementation)

Cognitive participation involves the collective efforts of palliative care staff to create networks of participation and communities of practice around the PCOC model [20].

### Sub-theme 3 familiarity and practical experience shifting attitudes

Clinicians initially resisted the PCOC model, but through training and continued engagement with quality report feedback, their attitudes gradually shifted as they saw its practical benefits and incorporated it into their routine work. One nurse stated, “*We were initially hesitant. After training*,* we began to accept it*,* but it didn’t become routine for the first three months. The quality report feedback sessions made us realize its benefits*,* and we sometimes discussed it with colleagues when we disagreed.*”

### Sub-theme 4 role of supportive networks in clinician engagement

Clinicians reported that it was crucial to have internal facilitators during the initial phase of implementing the PCOC model. The internal facilitators needed to familiarize themselves with the PCOC model and recognize its value before they could effectively encourage others to fully engage.

As one nurse recognized, “*There should be a staff member in charge of this program*,* and the person needs to be familiar with the program and recognize its value*,* then she will be willing to promote it.*”

Additionally, participants emphasized the importance of leadership, particularly from the clinical director of the palliative care unit. This was seen as a key factor in encouraging doctors and other healthcare providers to participate in the program.

Another nurse acknowledged, “*The clinical director is a key person to facilitate the PCOC model*,* the doctors will participate in this program with the involvement of the director*.”

Furthermore, clinicians stressed that support from management and colleagues, especially senior staff, was considered essential for the PCOC implementation. They also underscored the critical role of the IT department in embedding the PCOC tools into Electronic Medical Records (EMRs) to ensure seamless integration into clinical practice.

One nurse clearly identified that, “*Without the support from the IT department*,* by embedding the tools into our EMRs*,* we don’t believe we are willing to use the PCOC model.*”

### Sub-theme 5 challenges of a top-down implementation approach

The implementation approach significantly impacted clinicians’ attitudes towards the PCOC model. The top-down approach led to persistent negative attitudes as clinicians perceived the PCOC model as an imposed mandate that increased their workload without incorporating their perspectives. Consequently, the perceived value of the PCOC model fell below their expectation, and they reported a need for further modifications to their workflow to better facilitate the PCOC integration.

“*We thought the assessment procedures would be simplified with the PCOC tools. However*,* nothing changed*,* only extra assessment tools were added*,* it is a task imposed by management.*” Nurse.

### Collective action -(Integration of PCOC into clinical Practice)

Collective action refers to the work that clinicians do to integrate the PCOC model in their routine clinical practice [20]. They focused primarily on three areas: ensuring assessment accuracy; applying assessment results in clinical practice; and utilizing the three-monthly quality reports.

### Sub-theme 6 clinician collaboration on PCOC assessment accuracy

Clinicians reported they collaborated to enhance the precision of PCOC assessments by discussing discrepancies or uncertainties, ensuring that these assessments accurately captured the needs of patients and their families. As one nurse noted, “*We (nurses) sometimes discuss if the assessment scores for the patient are different between different nurses*”.

### Sub-theme 7 application of PCOC assessment results in clinical practice

The use of assessment information in clinical practice was multifaceted. Participants reported that they communicated the PCOC assessment results during handovers, relaying information from nurses to doctors, utilizing assessment results to guide conversations with patients and their families, and focusing on abnormal results to ensure prompt and appropriate responses. A nurse said, “*During morning handover*,* we update all staff on some patients’ conditions. And we also immediately report any abnormal scores to the doctor in charge.”*

### Sub-theme 8 utilization of PCOC quality reports for continuous improvement

The three-monthly PCOC quality reports were produced and used to conduct a reflective analysis of the care process, accompanied by an improvement plan. Clinicians found these reports valuable for identifying both areas where they excelled, such as pain management, and areas needing improvement, such as addressing the needs of families.

“*According to the last report*,* it indicated that we neglected the needs of families*,* yes*,* that’s true*,* we put our focus on patients… Pain management is what we did very well*.” Nurse.

Recognizing the value of the PCOC quality reports fostered a culture of continuous quality improvement and motivated further buy-in.

### Reflexive monitoring (Evaluation, refinement, and Sustainability)

Reflexive monitoring refers to how individuals and groups appraise the integration of the PCOC mode [20]. Within this construct, three themes emerged.

### Sub-theme 9 impact of the PCOC model on clinical work

Clinicians reported both positive and negative impacts on their daily work. Positive impacts aligned with the perceived value of the PCOC model, including enhanced symptom management, improved reflection on and improvement of the overall care processes, provision of a clear response framework that standardized clinical workflows and strengthened familiarity with patients and their families, leading to more personalized care. However, some still perceived it as an additional task mandated by management, resulting in an increased workload. As one nurse expressed, “*It did increase my workload*,* and I do it because it is required by our leader. Honestly*,* it feels more like a task to complete.*”

### Sub-theme 10 evaluation of PCOC implementation: challenges, necessity and success

Clinicians identified several challenges related to the user-friendliness of the PCOC model. They noted an overlap with existing assessment tools within their unit, which resulted in redundancy and increased workload. One nurse pointed out, “*Some PCOC assessment tools overlapped with the tools we are currently using*,* like RUG-ADL*,* we have Barth Index (BI)*,* as well as the pain item*”.

Additionally, components such as the AKPS and RUG-ADL were perceived as having limited clinical relevance. The subjective nature of some PCOC assessment tools posed difficulties in maintaining consistency across different clinicians. A doctor noted, “*Some item assessments are subjective*,* for example*,* AKPS*,* Palliative care phase*,* we need to have an agreement with nurses*,* further education is required.*”

Furthermore, the assessment frequency was seen as needing adjustment to better reflect the changing needs of patients and their families. These challenges, to some extent, negatively impacted clinicians’ attitudes toward the necessity and perceived success level of integrating the PCOC model into routine practice. One nurse stated that, “*In terms of the level of success of integrating the PCOC model into our practice*,* I would say it is a 6 out of 10*,* as you can see*,* we all put efforts into this*,* however*,* further adjustments are still needed.*”

### Sub-theme 11 recommendations for sustainability of the PCOC model

To ensure the sustainability of the PCOC model, several recommendations were made. It was suggested to align existing assessment measurements with PCOC tools to eliminate redundancy, adjust the assessment frequency to better capture patients’ and families’ needs, enhance the accuracy of assessments through continuous PCOC education and training, and streamline documentation processes. For example, one nurse suggested, “*I recommend cancelling the bedside document for the pain assessment and just keeping the record on the EMRs*.”

Integration of palliative care clinical guidelines into EMRs to assist clinicians in developing care plans based on assessment results and ensuring the implementation of these care plans were considered crucial in the application of PCOC assessment results. Other recommendations included incorporating the PCOC quality report into the hospital’s quality management systems. “*I suggest making the PCOC quality report part of the quality management indicators*,* then everyone will treat it seriously*”, one nurse recommended.

Furthermore, clinicians strongly recommended to enhance their knowledge and self-efficacy in palliative care through structured educational programs and to ensure the accessibility of medical resources in the hospital to support effective multidisciplinary collaboration. “*For example*,* when our patients need support from psychologists*,* there is one available for them to access*.” One doctor stressed, highlighting the importance of medical resources in delivering high-quality care.

## Discussion

This study employed an explanatory mixed methods approach to explore the implementation and integration of the PCOC model into a palliative care unit at a cancer hospital in Mainland China. Implementation outcomes demonstrated broad reach, high fidelity and sustained adoption, with qualitative findings explaining the mechanisms driving these trends. All inpatients (*n* = 355) were assessed using PCOC tools (Reach), reflecting clinicians’ recognition of its value in guiding patient care (Coherence). While initial clinician participation was 100%, it later stabilized at 75% (Adoption) due to staff leave or part-time work. Clinicians emphasized that leadership support and peer discussions reinforced engagement, while some perceived PCOC as an added workload (Cognitive participation). Fidelity remained high, with only 2.2% of daily assessments missing, supported by EMR integration and quality feedback sessions that helped standardize assessments (Collective action). However, clinicians noted that workflow misalignment and redundant documentation needed refinement for sustained adherence (Reflexive monitoring).

In integrating the PCOC model into routine clinical practice, we employed a “top-down” strategy, which was crucial for introducing this model to Mainland China. However, some negative attitudes toward the PCOC model persisted among clinicians after six months of implementation. They perceived that the model was imposed by leadership without clinical engagement and consultation. Consequently, the clinician perspectives, which could have realistically enhanced the implementation were not incorporated in the initial implementation. This included the adjustment of assessment frequency and the overlap of assessment tools within the unit. These negative attitudes made clinicians less motivated or less willing to use the PCOC model. Previous studies have supported the notions that a “top-down” approach could increase alignment and implementation speed but might reduce a sense of professional autonomy. Conversely, a “bottom-up” model can increase engagement and innovation but may threaten the fidelity of the program [27, 28]. Therefore, to achieve a balanced and effective integration, it is crucial to leverage the strengths of a “top-down” strategy for initial alignment and rapid implementation. Following this, it is essential to foster commitment and motivation from the “bottom” by actively engaging clinicians in the process. This dual approach ensures that the intervention is not only adopted but also sustained successfully in a new context.

To encourage clinicians to continue integrating the PCOC model, demonstrating its effectiveness in improving patient outcomes and enhancing the quality of care within their unit is essential. Bradshaw et al., also highlighted that the successful implementation of PCOMs requires continuous reinforcement of their value and effectiveness to clinicians [29]. In our study, clinicians highlighted three key areas to focus on to promote the effectiveness of this model. First, PCOC assessments should accurately and promptly reflect the needs of patients and their families. Second, response to these assessments must be timely and appropriate. Lastly, periodic review of the quality report, development and implementation of improvement plans to address the deficiencies identified in the PCOC quality report needs to occur. These are fundamental to leveraging the full potential of the PCOC model in clinical practice.

A major barrier to sustained implementation was workload concerns. Clinicians perceived PCOC as an additional documentation burden, similar to findings from studies on implementing patient-reported outcome measures (PROMs) [30]. To address these concerns, clinicians in our study suggested several strategies: aligning the PCOC model with the existing workflows, adjusting current assessment measures with PCOC tools to eliminate duplication, and streamlining documentation by integrating records of interventions implemented in response to PCOC assessments with nursing notes to avoid redundancy [31].

Additionally, clinicians reported that daily assessments of patient’s dependency and performance were considered to have limited clinical relevance, leading to unnecessary workload. This concern likely stems from a misunderstanding of the tools’ purpose, as the AKPS and the RUG-ADL were primarily viewed as mechanisms for allocating resources [32, 33]. However, beyond resources allocation, the AKPS also serves as a valuable predictor of patients’ outcome, such as prognosis, and can aid in making informed decisions that align with the goals of the patient and their family [32]. To address this gap in understanding, further training focused on clarifying the multifaceted purpose of these assessment tools is warranted to ensure clinicians fully appreciate their role in both patient care and resource management. Previous research has also highlighted the importance of ensuring clinicians understand when and why to use the assessment tool [29, 34].

Another noteworthy finding from this study, which has not been previously reported, is the recommendation for increased accessibility to palliative care educational programs and resources supporting multidisciplinary collaboration. In regions or countries where palliative care is less developed, it is crucial to enhance clinicians’ knowledge and self-efficacy in palliative care, as it could boost their confidence and better equip the delivery of holistic care for patients, including the application of person-centred outcomes-based quality improvement programs. Equally important is ensuring accessibility to a cross-section of health professionals including psychologist, nutritionists, therapists, to support interdisciplinary collaboration. These priorities are crucial for responding to patients’ and family needs and ensuring the quality of palliative care [35].

### Strengths and limitations

A key strength of this study is its mixed methods design, which combined quantitative outcome evaluation with qualitative process exploration. This approach allowed for a comprehensive understanding of the PCOC implementation, capturing what was achieved as well as how it occurred. By integrating both perspectives, the study enhances the practical relevance of its findings, offering valuable insights for future scale-up in similar healthcare settings.

However, this study has some limitations. First, the PCOC model was implemented in a single cancer hospital, which may limit the generalizability of findings to other healthcare settings. While this study provides valuable insights into the implementation of PCOC in a hospital-based palliative care unit, future research should expand to multiple palliative care settings, including community-based services and nursing homes. Additionally, the process evaluation was conducted after six months of PCOC implementation, constrained by the limited timeline of the PhD program. Conducting a longitudinal process evaluation at multiple points, such as six months and twelve months, would allow palliative care units time to further adapt to the model, adjust their perceptions, and provide deeper insights into its integration into routine practice.

### Conclusions and recommendations

**Fig. 1:**
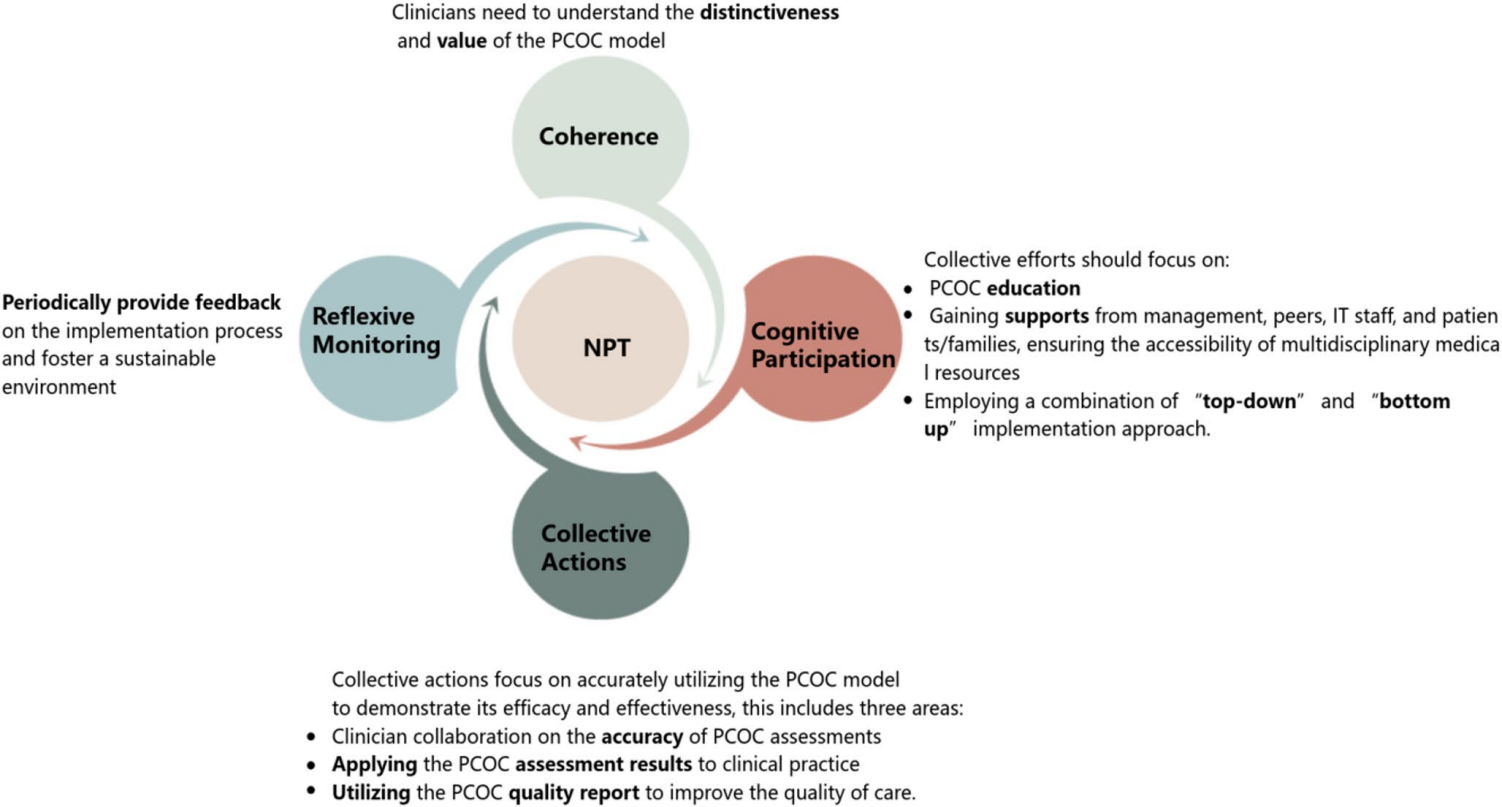
Recommendations for future implementation and integration of the PCOC model

Guided by NPT, we comprehensively evaluated the normalization of the PCOC model within a hospital-based palliative care unit in Mainland China. Recommendations for future implementation were developed based on clinician insights gathered through interviews and synthesized with study findings using NPT framework. A phased approach, combing an initial “top-down” approach followed by a “bottom-up”, was considered essential for aligning the program with the local context. Additionally, effectively applying the program to clinical practice without unnecessarily increasing the workload while simultaneously meeting stakeholders’ expectations is key to the sustainability of the program. Importantly, in settings with less developed palliative care, enhancing clinicians’ palliative care knowledge and self-efficacy and providing resources to support multidisciplinary collaboration are priorities. Figure 1 outlines recommendations derived from this study.

## Electronic supplementary material

Below is the link to the electronic supplementary material.

Supplementary Material 1

Supplementary Material 2

Supplementary Material 3

## Data Availability

The datasets used and/or analysed during this study are available from the corresponding author on reasonable request.
