# Supplementary material for: Process evaluation of a person-centred outcome measures-based quality improvement program in a hospital-based palliative care in mainland China
Source: Qual Life Res. 2025 May 27;34(9):2629–39. doi: 10.1007/s11136-025-03997-w (PMC12432087; doi:10.1007/s11136-025-03997-w)
Supplement: Supplementary file 2 — Supplementary Material 2 [file 11136_2025_3997_MOESM2_ESM.docx]

**
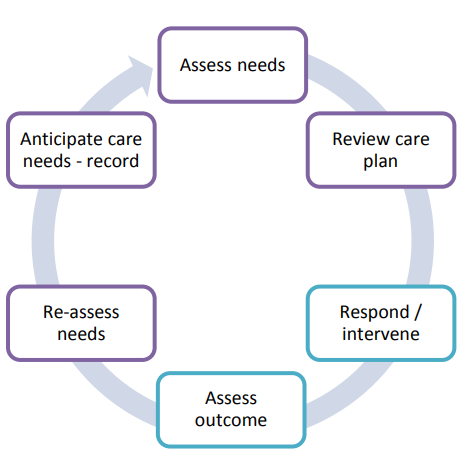
**

**Supplementary Figure 1 PCOC routine clinical assessment and response framework**

Note: The palliative care needs of patients are assessed using five standardized tools:

- PCOC Symptom Assessment Scale (PCOC-SAS) for symptom distress;
- Palliative Care Problem Severity Score (PCPSS) for symptom severity;
- Palliative Care Phase for clinical acuity and urgency;
- The Australia-modified Karnofsky Performance Status (AKPS) for performance status;
- The Resource Utilization Groups - Activities of Daily Living (RUG-ADL) for functional dependency.

**
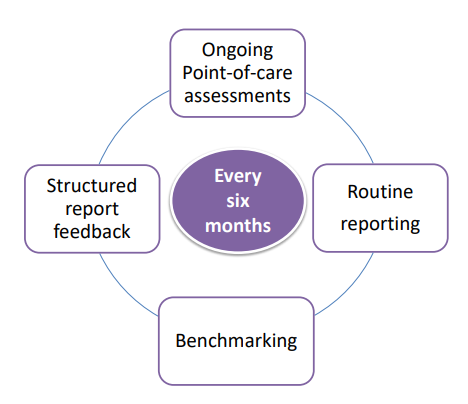
**

**Supplementary Figure 2 PCOC cycle**

**Supplementary table 1 Specification of the PCOC model implementation strategies**

| **Implementation stages** | **ERIC categories** | **ERIC implementation strategies** | **Specification of the implementation strategies** |
| --- | --- | --- | --- |
| **Pre-implementation** | Train and educate stakeholders | Conduct educational meetings | Actor: External facilitators  Action:   - PCOC fundamental education sessions - On-site case studies   Action target: Hospital leaders, palliative care managers, and palliative care clinicians  Justification: Employed a “top-down” approach to demonstrate the benefits of the PCOC mode to leaders, managers and clinicians, encouraging their buy-in. To equip palliative care clinicians with knowledge of the PCOC model |
|  |  | Distribute educational materials | Translated the PCOC clinical manual from English to Chinese, and distributed it to palliative care clinicians |
|  | Adapt and tailor to context | Tailor strategies | Actor: External facilitators, hospital leaders, and palliative care managers  Action:   - Overcame the identified barriers and leveraged facilitators by incorporating local knowledge - Conducted a scoping review to explore the strategies used to support implementation [28] - Utilized recommendations from the Australian PCOC centre (Implementation strategies document) [6] |
|  |  | Promote adaptability | Actor: Hospital leaders, palliative care managers  Action: Adjusted the assessment frequency of the PCOC model based on the local context. The frequency was set to once daily, a modification from the original PCOC recommendation of “at least once a day” based on changing needs  Justification: To avoid increasing the workload |
|  | Use evaluative and iterative strategies | Identify barriers and facilitators | Actor: External facilitators  Action: Conducted focus group interviews and in-depth individual interviews with hospital leaders, palliative care managers, and palliative care clinicians  Justification: To develop tailored implementation strategies based on identified barriers and facilitators |
|  |  | Conduct local need assessment | Actor: External facilitators  Action:   - Developed a dashboard for PCOC assessment in Electronic Medical Records (EMRs) - Added Delirium and Survival Days Prediction assessments for palliative care patients   Action target: Palliative care managers and clinicians  Justification:   - To create a dashboard that meets clinicians’ preferences - To enable comprehensively assess the needs of palliative care patients - To guide clinicians in accurately assess the Terminal Phase |
|  |  | Stage implementations scale up | Actor: Palliative care clinicians  Action: Conducted PCOC assessments for two months prior to formal implementation  Justification:   - To cross-culturally adapt and validate the PCOC assessment tools - To gain familiarity with the PCOC model |
|  | Provide interactive assistance | Provide local technical assistance | Actor: External facilitators, hospital leaders, IT staff, palliative care managers and clinicians  Action: Embedded the PCOC tools and palliative care clinical guidelines into EMRs  Justification:   - To develop a digital data collection system - To guide clinicians’ responses to assessments by embedding clinical guidelines into the EMRs as clinicians, addressing reported gaps in palliative care knowledge and self-efficacy |
|  | Develop stakeholder interrelationships | Use an implementation advisor | Actor: An experienced Australian palliative care clinical director  Action: Shared her experience of using the PCOC model in her routine work  Action target: Palliative care managers and clinicians  Justification: To encourage Chinese palliative care professionals to buy-in the PCOC model |
|  |  | Identify and prepare champions | Identified and prepared the nurse manager and a senior nurse as internal facilitators |
|  |  | Involve executive boards | Supports from the Nursing department |
|  | Change infrastructure | Mandate change | Actor: Hospital leaders, palliative care managers  Action:   - Assessed patients daily using the PCOC tools - Kept records of the PCOC assessments and corresponding interventions - Communicated the assessments during handovers   Action target: Palliative care clinicians |
|  |  | Change record systems | Actor: Hospital leaders, palliative care managers  Action: Kept records of interventions based on the PCOC assessment results  Action target: Palliative care clinicians |
| **Implementation** | Use evaluative and iterative strategies | Audit and provide feedback | Actor: External facilitators  Action:   - Checked the PCOC assessment data - Provided three-monthly quality report and improvement plan   Action target: Palliative care managers and clinicians  Justification:   - To ensure accurately utilization of the PCOC tools - To provide feedback on the quality of palliative care - To identify areas in needs of improvement |
|  | Train and educate stakeholders | Provide ongoing consultation | Actor: External facilitators  Action: Provided online support  Action target: Palliative care managers and clinicians |
|  |  | Conduct ongoing training | Actor: External facilitators  Action: Provided PCOC advanced education  Action target: Palliative care managers and clinicians |
|  | Support clinicians | Facilitate relay of clinical data to providers | Actor: Palliative care nurses  Action: Reported abnormal PCOC assessments to doctors  Action target: Palliative care doctors  Justification: To guide clinical practice using the PCOC assessments |
|  |  | Remind clinicians | Actor: Internal facilitators  Action:   - Checked the completion of the PCOC assessments - Provided the PCOC response framework at nursing station and doctors’ office   Action target: Palliative care clinicians |

**Supplementary table 2 The question guideline for the qualitative component**

| **NPT Constructs** | | **Questions** |
| --- | --- | --- |
| **Coherence** | Differentiation | 1.Please describe your experience of being involved in the implementation and integration of PCOC in your ward from the very beginning to the present? Such as your knowledge, attitude, expectation regarding to the PCOC program. How has this influenced the implementation and integration of PCOC into the daily practice?  2.How did the doctors and the nurses feel about the implementation and integration of PCOC during this period? (Including how they feel about it today)  3.What value do you think the PCOC program has so far? Is the value of the PCOC as you expected？  4.In your opinion, how does the PCOC program differ from other quality improvement programs? |
|  | Individual specification |  |
|  | Communal specification |  |
|  | Internalization |  |
| **Cognitive Participation** | Initiation | 5.How necessary do you think integrate the PCOC into your daily practice?  6.Please help me understand how PCOC is integrated into your daily practice by sharing some examples of how PCOC forms part of your daily work?  **# Prompts:** If you still don’t think PCOC is a part of your daily work so far, why?  7.Is there a key person responsible for facilitating the utilization of the PCOC program in your daily practice? What have they done to promote its implementation and integration? |
|  | Legitimation |  |
|  | Enrolment |  |
|  | Activation |  |
| **Collective Action** | Interactional workability | 8.How have the PCOC assessment results been used to guide adjustments in the care plan for the inpatients?  **# Prompts:** If the PCOC assessment results have not been used to guide adjustments in the care plan for the inpatients, why?  9.How do you identify areas that need improvement by using the PCOC quality report?  10.Who should be involved in identifying the areas that need improvement by analyzing the PCOC quality report and making quality improvement strategies?  11.What supports have you been received from the hospital and/or department to facilitate the implementation and integration of the PCOC program from its inception to the present? |
|  | Skillset workability |  |
|  | Relational integration |  |
|  | Contextual integration |  |
| **Reflexive Monitoring** | Systematization | 12.What feedback have your received regarding the implementation and integration of PCOC into the routine practice?  13.What adjustments did you make after receiving feedback regarding the implementation and integration of the PCOC? What adjustments did other clinicians at your ward make?  14.How has PCOC impact your work?  15.What other suggestions for sustaining the PCOC program in your ward or advice for other palliative care unit to better adopt PCOC in the future? |
|  | Individual appraisal |  |
|  | Communal appraisal |  |
|  | Reconfiguration |  |
|  |  | 16.In your opinion, how successful do you think the implementation and integration of the PCOC program into your daily practice？From a scale of 1 to 10, where 1 is unsuccessful, and 10 is successful.  17.Is there anything else you would like to add to our conversation today? |

**Supplementary table 3 Using NPT to evaluate the integration of the PCOC model in hospital-based palliative care unit ─ a mixed study**

| **CMO domain** | **NPT construct** | **Description and results** |
| --- | --- | --- |
| **Implementation contexts** |  | **Description:** *Contexts are patterns of social relations and structures that unfold over time and across setting. They make up the implementation environment.*  The detailed information on the local context and the modifications/adjustments for the PCOC model and the workflow are presented in the Methods section (refer to Supplementary table 1). |
| **Implementation mechanisms:**  Mechanisms are revealed through purposive social action ─ **collaborative work** ─ that involves the investment of personal and group resources to achieve goals. | **Coherence building** | **Description:** *How do people work together in everyday settings to understand and plan the activities that need to be accomplished to put the PCOC model into practice?*  **Results:**   - **Sub-theme 1-Distinctiveness of PCOC compared to other quality initiatives:** - Different from satisfactory survey, the PCOC could identify the areas that need to be improved. - Objectively assess quality of care from the patient’s perspective. - Strengthen cooperation between doctors and nurses. - Forming a closed loop cycle of quality management. - Response framework to assessment results. - Comprehensive assessments. - Qualification of care quality. - **Sub-theme 2-Perceived value of the PCOC model in patient care:** - Providing timely and personalized care for patients. - Assisting in the formulation of medical decisions and care plans. - Improving communication between clinicians and patients/families, between doctors and nurses. - Clarifying the content of clinical work. - Establishing a patient-centered care quality system in China. |
|  | **Cognitive participation** | **Description:** *How do people work together to create networks of participation and communities of practice around the PCOC model?*  **Results:**   - **Sub-theme 3-Familiarity and practical experience shifting attitudes:** - Enhance clinicians’ understanding and attitudes toward the PCOC model through targeted education sessions. - **Sub-theme 4-Role of supportive networks in clinicians’ engagement:** - Role of internal facilitators: - Becoming familiar with the PCOC model. - Recognizing the benefits of the PCOC model. - Leadership: Support from the clinical directors and nurse managers. - Broad support networks: - Support from the management and colleagues, particularly senior staff. - Support from the IT department. - Involvement of patients and their families. - **Sub-theme 5-Challenges of a top-down implementation approach:** - The top-down approach without incorporating clinicians’ perspectives, leading to persistent negative attitude towards the PCOC model: - Perceived as a Top-Down mandate, viewed as an increase in workload. - The value of the PCOC falls below their initial expectation. - Further modifications required to fit the PCOC to the local context. |
|  | **Collective action** | **Description:** *How do people work together to enact the PCOC model?*  **Results:**   - **Sub-theme 6-Clinician collaboration on PCOC assessment accuracy:** - Clinicians discuss PCOC assessments to enhance their precision, ensuring that they accurately capture the needs of patients and their families. - **Sub-theme 7-Application of PCOC assessment results in clinical practice**   Methods of application:   - Handover communication: Communicate PCOC assessment results during handover. - Information relay: Nurses relay assessment results to doctors for informed decision-making. - Guided conversations: Utilize results to guide conversations with patients and their families. - Attention to abnormalities: Allocate increased attention to abnormal assessment results to ensure prompt and appropriate responses. - **Sub-theme 8-Utilization of the PCOC quality report for continuous improvement**   Method of application:   - Reflective Analysis: Utilize the PCOC quality report to conduct a reflective analysis of the care process, identifying both strengths and areas in need of improvement to continually enhance patient care. |
|  | **Reflexive monitoring** | **Description:** *How do people work together to appraise the PCOC model?*  **Results:**   - **Sub-theme 9-Impact of the PCOC model on clinical work** - Positive impacts: - Enhanced symptom management: Improved focus on managing patients’ symptoms by doctors. - Care process improvement: Increased reflection on and improvement of the overall care process. - Workflow standardization: Provision of clear guidelines that standardize clinical workflows. - Strengthened patient relationships: Enhanced familiarity with patients, leading to more personalized care. - Negative impacts: - Increased workload: Perception of the PCOC model as an additional task mandated by management, leading to an increased workload for clinicians. - **Sub-theme 10-Evaluation of PCOC implementation: challenges, necessity and success** - **Challenges in applying PCOC in practice** - Overlap with the existing tools: The PCOC tools overlap with some pre-exiting assessment tools, leading to redundancy. - Limited clinical relevance: AKPS and RUG-ADL have limited applicability in clinical practice. - Subjective: The PCOC assessment tools are subjective, making it difficult to ensure consistency in assessments among different clinicians. - Dynamic assessment frequency: The frequency of assessment should be dynamic to reflect the changing needs of patients/families. - **Necessity of integrating the PCOC model into routine clinical practice** (scale: 0 – 10, 0 = Unnecessary at all, 10 = Highly necessary)**:** - Score 5-6: Reported by five clinicians. Reasons: - Lack of dynamic assessing (negative). - AKPS and RUG-ADL are not meaningful for clinical guidance (negative) - Necessary but need further modifications to fit in with the local context (positive). - Score 7: Reported by one clinician. Reasons: - Continuously track the needs of patients. - Score 9-10: Reported by two clinicians. Reasons: - As important as measuring daily vital signs for patients. - Qualification of care quality. - **Assessment of PCOC implementation success** (scale: 0 – 10, 0 = Not successful at all, 10 = Most successful) - Score 5-6: Reported by three clinicians. Reasons: - Daily conduct of PCOC assessments (positive). - Limited level of doctor’s participation (negative). - Negative attitudes toward the PCOC model (negative). - Overlap of some PCOC assessment tools with existing tools, requiring further adaptation to the local context (negative). - Doubts about the overall value of the PCOC model (negative). - Score 7-8: Reported by six clinicians. Reasons: - Daily conduct of PCOC assessments (positive). - Recognition of the importance of PCOC assessment (positive). - Need to further increase the accuracy of PCOC assessment (negative). - Lack of dynamic assessment frequency (negative). - Insufficient application of PCOC assessment data in clinical practice (negative). - Score 9-10: Rated by two clinicians. Reasons: - Integration of PCOC assessment into routine patient assessments, similar to “vital signs” monitoring (positive). - Quantification and enhancement of care quality through PCOC assessments (positive). - **Sub-theme 11-Future recommendations for long-term sustainability** - Regarding PCOC Assessment and Recording: - Eliminate redundancy: Align the exiting assessment measurements with PCOC tools to avoid duplication. - Dynamic needs assessment: Conduct dynamic assessments of patients' and families' needs as they evolve. - Enhance precision: Enhance the accuracy of assessments through continuous PCOC education and training. - Streamline documentation: Integrate PCOC assessment records with nursing documentation to prevent redundancy. - Regarding the application of PCOC assessment results: - Integrate clinical guidelines: Incorporate palliative care clinical guidelines into EMRs to assist clinicians in developing care plans based on PCOC assessments. - Team collaboration: More involvement of doctors. - Address patient/family needs: Implement care plans on PCOC assessment results to effectively meet patient’s/family’s care needs. - Regarding PCOC quality reports: - Quality management integration: Incorporate the PCOC quality report into the hospital’s quality management system. - Development and implementation of improvement plans: Developing plans that address the deficiencies identified in the PCOC quality report. And ensure these plans are not only created but also effectively implemented to achieve improvements in care delivery. - Other recommendations: - Educational programs: Enhance clinicians’ knowledge and self-efficacy in palliative care through structured educational programs. - Resource accessibility: Ensure the accessibility of medical resources to support effective multidisciplinary collaboration. |
| **Implementation outcomes:**  The practical effects of implementation mechanisms at work | **Intervention performance** | **Description:** *What practices have changed as the result of the PCOC model being operationalized, enacted, reproduced, over time and across settings?*  **Results:**   - **Reach:** 100% (n=355) palliative care inpatients were assessed by the PCOC tools during the six months of PCOC implementation. - **Adaptions:** Twelve out of 16 clinicians in the palliative care unit participated in the PCOC model. (Initially, all clinicians were involved starting in June 2023. However, three later went on maternity leave, and one opted out.) - **Fidelity:** - Tools assessment: - Assessment frequency: The five PCOC tools were administered once a day. - Rate of data item completion: 97.8% (3134/3205 daily assessments) - Quality feedback reports and improvement plan: Two three-monthly quality feedback reports were produced, each accompanied by an improvement plan to address identified areas needing enhancement. |
|  | **Relational restructuring** | **Description:** *How have working with the PCOC model changed the ways people are organized and relate to each other?*  **Results:** N/A |
|  | **Normative restructuring** | **Description:** *How has working with the PCOC model changed the norms, rules and resources that govern action?*  **Results:** N/A |
|  | **Sustainment (normalization)** | **Description:** *How has the PCOC model become incorporated in practice?*  **Results:** Please refer to Supplementary table 1 Specification of the PCOC model implementation strategies. |
